# The APOE Genotype in Idiopathic Normal Pressure Hydrocephalus

**DOI:** 10.1371/journal.pone.0158985

**Published:** 2016-07-21

**Authors:** Yi Yang, Mats Tullberg, Kirsten Mehlig, Annika Rosengren, Kjell Torén, Henrik Zetterberg, Carsten Wikkelsö

**Affiliations:** 1 Institute of Neuroscience and Physiology, Sahlgrenska Academy, University of Gothenburg, Gothenburg, Sweden; 2 Department of Public Health and Community Medicine, Sahlgrenska Academy, University of Gothenburg, Gothenburg, Sweden; 3 Department of Molecular and Clinical Medicine, Sahlgrenska Academy, University of Gothenburg, Gothenburg, Sweden; 4 Section of Occupational and Environmental Medicine, Institute of Medicine, Sahlgrenska Academy, University of Gothenburg, Gothenburg, Sweden; 5 Occupational Medicine, Respiratory Diseases and Toxicology, University of Perugia, Perugia, Italy; 6 Institute of Neuroscience and Physiology, Department of Psychiatry and Neurochemistry, Sahlgrenska Academy, University of Gothenburg, Gothenburg, Sweden; 7 UCL Institute of Neurology, Queen Square, London, United Kingdom; Torrey Pines Institute for Molecular Studies, UNITED STATES

## Abstract

**Introduction:**

Amyloid plaque has been reported in brain biopsies from patients with idiopathic normal-pressure hydrocephalus (iNPH) and proposed as a significant feature of the pathophysiology. Presence of the apolipoprotein E ε4 (*APOE* ε4) allele is associated with increased risk of Alzheimer’s disease (AD).

**Aims:**

To compare the distribution of *APOE* genotype in iNPH patients with an age-matched population-based control group and with Alzheimer’s disease (AD) patients.

**Methods:**

*APOE* genotype frequencies were determined in 77 iNPH patients (50 men and 27 women, mean age 71.7 years) diagnosed with iNPH, a sample of 691 AD patients and 638 age-matched population controls (299 men and 339 women) from the INTERGENE cohort.

**Results:**

The *APOE* distribution did not differ significantly between the iNPH patients and the control population. The per e4-allele odds-ratio (OR) of iNPH was given by OR = 0.90, 95% confidence interval (CI) = (0.50, 1.60) that was considerably smaller than the per-allele OR of AD, OR = 5.34 (4.10, 7.00).

**Conclusion:**

The results suggest that the APOE-related risk of AD in patients with iNPH is not higher than in the general population.

## Introduction

A high occurrence of Alzheimer’s disease (AD) among patients with idiopathic Normal Pressure Hydrocephalus (iNPH) has been reported[1–3] which has led to speculation that iNPH and AD share a common pathophysiological background[4]. Accumulation of amyloid-β plaques is one of the fundamental characteristics of AD. Apolipoproteins enhance the breakdown of amyloid-β. However, the E4 isoform APOE is ineffective and leads to amyloid build-up in the brain [5] The presence of the E4-encoding *APOE* ε4 allele increases the risk of AD by 3 times in heterozygotes and by 15 times in homozygotes [6]. Amyloid plaques have been found in cortical brain biopsies from NPH^7^ as well as iNPH^8^ patients. Leinonen et al [7] found a significant correlation between positive biopsies and AD co-morbidity among NPH patients while Bech-Azeddine et al [8] found no association between biopsies and clinical presentation of AD among iNPH patients. Silverberg et al [4] suggested that CSF stagnation in iNPH leads to accumulation of extracellular amyloid-β42 leading to increased risk of AD. Hypothetically, accumulation of amyloid-β aggregates in the choroid plexus and the arachnoid membranes leads to reduced CSF absorption and communicating normal-pressure hydrocephalus.

The aim of this study was to compare risk factors for iNPH and AD, specifically, whether the *APOE* ε4 allele is more frequent among iNPH patients than among population controls. For comparison, similar analyses were performed in a sample of patients with AD.

## Patients and Methods

### Patients with iNPH

One hundred and eight patients (mean age 71.9 years, SD 8.9 years, range 40–89 years), 65 men and 43 women, consecutively diagnosed with probable iNPH according to the American-European guidelines [9] at the Hydrocephalus Research Unit during the years 2006–2011 were included.

All patients had symptoms and signs scored by the iNPH scale [10] before and after surgery. A change in the score of ≥ 5 points was categorized as good outcome and < 5 points as poor outcome.

All were operated upon by an adjustable ventriculo-peritoneal shunt (PS Medical Strata®, Medtronic Inc., Goleta, CA, USA or Codman & Schurtleff / Johnson & Johnson Co., Raynham, MA, USA).

Thirty-one patients, 15 men and 16 women, had no blood sample taken, leaving 77 patients (mean age 71.7, SD 8.7, range 48–87 years), 50 men and 27 women, for genotyping. Six of these patients had no outcome scores.

### Patients with AD

Eight hundred and three patients of Swedish nationality diagnosed with AD from a study by Zetterberg et al [11] were included for comparison with iNPH patients and the INTERGENE control population. Six hundred ninety one patients (mean age 76.2 years, SD 6.9 years, range 50–102 years), 259 men and 432 women had data for relevant variables and were included in this study. The patient population and methods are described in the original article.

### Blood samples

Genomic DNA was isolated from EDTA whole blood and *APOE* (gene map locus 19q13.2) genotyping was performed by mini-sequencing as described previously in detail [12] Genotypes were obtained for the two single nucleotide polymorphisms that are used to unambiguously define ε2, ε3, and ε4 alleles (rs7412 and rs429358).

### Reference population

The INTERGENE cohort (http://www2.sahlgrenska.gu.se/intergene/eng/index.jsp) consists of a population-based random sample of inhabitants of Gothenburg and the surrounding region of Västra Götaland (South-west Sweden) and between 25 and 75 years old at the time of sampling [13]. Between April 2001 and December 2004, 3614 members of the target population sample were examined (participation rate 42%). Genotyping was successful in 3204 subjects (1524 men, 1680 women), and 638 subjects ≥ 65 years at the time of examination (mean age 69.9 years, SD 3.0 years, range 65–77 years), 299 men and 339 women, were included as control subjects for iNPH patients in this study. The lower age limit was chosen such that the average age was similar in cases and controls, to reduce the probability that cases were compared to younger controls that would develop the disease had they reached the age of the patients.

All subjects gave their written consent to the study, and the protocol was approved by the regional ethics review board, Ethics Committee at the University of Gothenburg (Ö 237/2000). All personal identifiers were protected by replacing them with a code. The study complies with the Declaration of Helsinki.

### Ethics

This study has been approved by the regional ethics review board in Gothenburg, registration number 154–05.

### Statistical analysis

The chi-square test was used to compare the overall distribution of genotype in iNPH and AD patients with the distribution in control subjects. The hypothesis of a higher proportion of cases for a higher number of Apo-E ε4 allele was tested using the Cochrane-Armitage test of trend. Using logistic regression, the effect size was estimated in terms of an odds ratio (OR) of iNPH or AD per e4 allele with 95% confidence interval (CI), adjusted for age, a quadratic term for age, and sex.

To test whether the three alleles were in Hardy-Weinberg equilibrium (HWE) we calculated the expected numbers of genotypes from product terms of (p_2_ + p_3_ + p_4_)^2^ times the total number of genotypes, with p_2_-p_4_ indicating the observed prevalence of each allele. Observed and expected counts were compared by a chi-square test with 3 degrees of freedom.

Age, sex, preoperative iNPH scale score and postoperative improvement was compared between participant and non-participant iNPH patients using the two-sample t-test and chi-square test for continuous and binary variables, respectively. Among participants, the association between preoperative iNPH score and the number of ε4 allele was investigated using linear regression, with and without adjustment for age and sex.

Statistical analyses were performed using IBM SPSS 17.0 for Windows (IBM, Armonk, NY, USA). The level of significance was set to 0.05 (2-sided tests).

## Results

### ApoE distribution and iNPH

**Fig 1:**
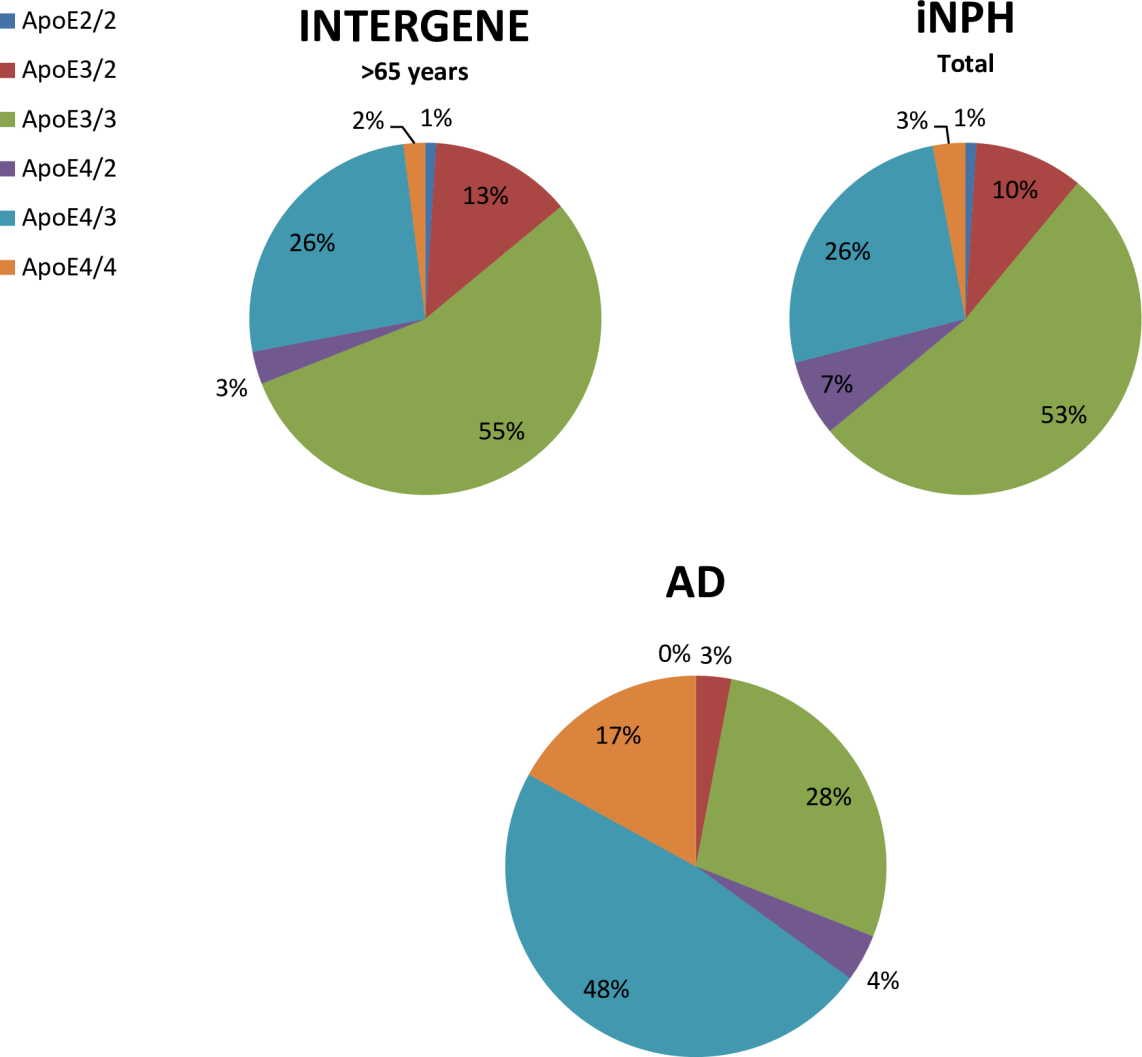
Pie chart of apolipoprotein E distribution in patients and control groups.

The overall genotype distribution in the 77 iNPH patients was similar to the distribution in the 638 controls (1^st^ part of Table 1, Fig 1). Among iNPH patients there was a higher proportion of genotypes with one or two ε4 allele compared to controls, however, the trend was not significant (2^nd^ part of Table 1). The trend of higher risk of iNPH for a higher number of ε4 allele was not significant when adjusted for age and sex using logistic regression (last part of Table 1). Forty-six patients (65%) were improved and 25 (35%) unchanged or deteriorated at the re-examination. There were no differences in *APOE* allele or genotype frequencies between iNPH patients with poor outcome and good outcome, compared to the control population (Table 1).

**Table 1:** *APOE* allele distribution among 77 iNPH patients and 691 AD patients, compared to 638 controls from INTERGENE. The genotypes were in Hardy-Weinberg equilibrium in all groups.

	INTERGENE (n = 638)	All iNPH (n = 77)	Poor outcome^a^ (n = 25)	Good outcome^a^ (n = 46)	AD (n = 691)
**Age** ^b^	69.9 (3.0)	71.7 (8.7)	72.1 (9.5)	70.9 (8.4)	75.8 (7.0)
**Female sex, n (%)**	339 (53%)	27 (35%)	9 (36%)	17 (37%)	432 (63%)
***APOE* genotype frequency, n (%)**					
*ε2/2*	6 (1%)	1 (1%)	0 (0%)	0 (0%)	0 (0%)
*ε3/2*	83 (13%)	8 (10%)	3 (12%)	4 (9%)	23 (3%)
*ε3/3*	353 (55%)	41 (53%)	13 (52%)	26 (56%)	194 (28%)
*ε4/2*	17 (3%)	5 (7%)	1 (4%)	4 (9%)	25 (4%)
*ε4/3*	166 (26%)	20 (26%)	7 (28%)	11 (24%)	331 (48%)
*ε4/4*	13 (2%)	2 (3%)	1 (4%)	1 (2%)	118 (17%)
**p-value (Χ**^**2**^**-test)**	Ref.	*0*.*6*	*1*.*0*	*0*.*3*	*<0*.*001*
**ε4 allele frequency, n (%)**					
*0*	442 (69%)	50 (65%)	16 (64%)	30 (65%)	217 (31%)
*1*	183 (29%)	25 (32%)	8 (32%)	15 (33%)	356 (52%)
*2*	13 (2%)	2 (3%)	1 (4%)	1 (2%)	118 (17%)
**p-value (trend)**	Ref.	*0*.*4*	*0*.*5*	*0*.*6*	*<0*.*001*
**Prevalence of ε4** ^c^	0.16 (0.14–0.18)	0.19 (0.13–0.25)	0.20 (0.08–0.32)	0.18 (0.11–0.26)	0.43 (0.40–0.45)
**OR** ^d^	Ref.	0.90 (0.50–1.60)	0.79 (0.19–1.65)	0.93 (0.45–1.91)	5.34 (4.10–7.00)

^a^ Numbers for good/poor outcome do not sum up to total number of iNPH patients due to missing values in postoperative iNPH score.

^b^ Data are given as mean values and standard deviation (SD).

^c^ Brackets show 95% CI.

^d^ OR (95% CI) for iNPH or AD, respectively, and an increase by one ε4 allele, adjusted for age, age^2^, and sex.

### ApoE distribution and AD

In patients with Alzheimer’s disease, the prevalence of ApoE 4 allele was significantly higher than in controls, and the age- and sex-adjusted OR indicated a more than five-fold risk of AD per e4 allele (last column in Table 1). All differences were equally significant in comparison with iNPH patients (data not shown).

## Discussion

The *APOE* ε4 allele represents a major risk factor for AD in all ethnic groups, across all ages between 40 and 90 years, and in both men and women [14]. In this population of iNPH patients the distribution of *APOE* alleles and genotypes was similar to that in a control population of predominantly healthy individuals, thus giving no support to a common pathophysiological mechanism in iNPH and AD. This paper adds important knowledge to previous reports due to the study design with a large sample of iNPH patients consecutively diagnosed and evaluated according to current guidelines being compared to a large cohort of patients with AD and a population-based random sample of controls.

Bech-Azeddine et al reported AD associated changes in 7 of 28 brain biopsies taken from iNPH patients and suggested a possible overlapping pathophysiology between iNPH and AD[8]. However they included 12 patients with AD co-morbidity and 17 with cerebrovascular disorder among the 28 iNPH patients indicating that the high occurrence might be due to the mixed study population and lack of strict inclusion criteria. Unexpectedly they found no correlation between the clinical diagnoses of possible AD (12 patients) and the analyses of the cortical biopsies. Only three out of 12 with possible AD showed AD pathology in the brain biopsies. Further, the presence of AD co-morbidity, or AD changes in the brain biopsies did not preclude clinical improvement after shunt operation. As could be expected the outcome was poor compared to other studies [15] with an improvement rate of only 33%.

Frontal cortical biopsies were taken and analysed for amyloid β (Aβ) and hyperphosphorylated tau (Pt) in 433 patients with presumed iNPH examined by Leinonen et al [7]. Two-hundred and nineteen were shunted of which 70 had Aβ with or without Pt in the biopsy samples. Of these 52 improved after shunting. At the retrospective diagnostic follow up 94 patients were diagnosed as AD of which 82 had Aβ with or without Pt in the biopsy samples. Nineteen of the 82 had been shunted based on ICP examinations with good outcome in 13 patients. The last finding might suggest a common pathophysiological mechanism and suggest clinical trials treating AD patients with shunts. However, none of the studies included controls and the evaluation of the biopsies was not blinded.

Silverberg and others suggested that accumulation of amyloid plaques in iNPH could be caused by stagnation of CSF [4]. In parallel to the successful treatment of iNPH patients with CSF diverging devices Silverberg treated AD patients with shunts, but unfortunately without success [16].

Pyykko et al found in 202 presumed NPH patients that *APOE* ε4 genotype associated independently with the presence of Aβ plaques in frontal cortical biopsy and was neither a risk factor for iNPH nor a predictor of shunt responsiveness [17]. They also found the *APOE* ε4 genotype in iNPH similar to healthy individuals. Our results reported here confirm these findings and altogether strengthen the notion that in iNPH patients, there is not an increased risk of AD. Accordingly, although we did not analyze the association between APO E e4 genotype and outcome in cognitive or other specific symptom domains, we found no differences in *APOE* allele or genotype frequencies between iNPH patients with poor outcome and good outcome, compared to the control population.

Support for a specific iNPH pathophysiology different from AD was the recent finding of a non-AD CSF biomarker profile in iNPH patients [18]. In contrast to the isolated reduction in amyloid β42 in AD, all amyloid isoforms and amyloid precursor proteins were reduced in iNPH possibly indicating a reduced brain metabolism in the periventricular zone. Other studies on CSF biomarkers have shown similar differences [19,20].

The iNPH population presented here was carefully defined according to predefined criteria and representative of what is commonly seen in iNPH. Patients were examined by a neurologist, a physiotherapist and a neuropsychologist, and symptoms and signs of gait, balance, cognitive and urinary functions were scored by the iNPH scale before and after shunt surgery. The improvement rate was 65%, which is slightly lower than recent reports on outcome after shunt surgery but still acceptable [15]. The iNPH patients were included consecutively and there were no differences between those with *APOE* allele analyses and those without (the causes for not sampling blood were administrative).

The small sample size of iNPH patients with a small proportion of females was a major limitation. However, this limitation does not explain the observed non-association between Apoe 4 allele and iNPH, as the comparison between effect size between AD (OR = 5.3 per e4-allele) and iNPH (OR = 0.9) shows.

In conclusion, the distribution of Apo-E genotype among iNPH patients is similar to population controls and does not support an APO E associated increased risk for AD in iNPH patients.

## References

[1] Del Bigio MR, Cardoso ER, Halliday WC. Neuropathological changes in chronic adult hydrocephalus: Cortical biopsies and autopsy findings. Canadian Journal of Neurological Sciences. 1997;24(2):121–126. 9164688 10.1017/s0317167100021442

[2] Savolainen S, Paljärvi L, Vapalahti M, Borgesen SE. Prevalence of Alzheimer's disease in patients investigated for presumed normal pressure hydrocephalus: A clinical and neuropathological study. Acta Neurochirurgica. 1999;141(8):849–853. 10536721 10.1007/s007010050386

[3] Golomb J, Wisoff J, Miller DC, Boksay I, Kluger A, Weiner H, et al Alzheimer's disease comorbidity in normal pressure hydrocephalus: Prevalence and shunt response. Journal of Neurology Neurosurgery and Psychiatry. 2000;68(6):778–781. 10.1136/jnnp.68.6.778 PMC1736969 10811706

[4] Silverberg GD, Mayo M, Saul T, Rubenstein E, McGuire D. Alzheimer's disease, normal-pressure hydrocephalus, and senescent changes in CSF circulatory physiology: a hypothesis. Lancet Neurol. 2003;2(8):506–511. 12878439 10.1016/s1474-4422(03)00487-3

[5] Polvikoski T, Sulkava R, Haltia M, Kainulainen K, Vuorio A, Verkkoniemi A, et al Apolipoprotein E, dementia, and cortical deposition of β-amyloid protein. New England Journal of Medicine. 1995;333(19):1242–1247. 7566000 10.1056/NEJM199511093331902

[6] Blennow K, de Leon MJ, Zetterberg H. Alzheimer's disease. Lancet. 2006;368(9533):387–403. 16876668 10.1016/S0140-6736(06)69113-7

[7] Leinonen V, Koivisto AM, Savolainen S, et al Amyloid and tau proteins in cortical brain biopsy and Alzheimer's disease. Annals of Neurology. 2010;68(4):446–453. 10.1002/ana.22100 20976765

[8] Bech-Azeddine R, Høgh P, Juhler M, Gjerris F, Waldemar G. Idiopathic normal-pressure hydrocephalus: Clinical comorbidity correlated with cerebral biopsy findings and outcome of cerebrospinal fluid shunting. Journal of Neurology, Neurosurgery and Psychiatry. 2007;78(2):157–161. 17012342 10.1136/jnnp.2006.095117 PMC2077673

[9] Relkin N, Marmarou A, Klinge P, Bergsneider M, Black PM. Diagnosing idiopathic normal-pressure hydrocephalus. Neurosurgery. 2005;57(3 Suppl):S4–16; discussion ii-v. 16160425 10.1227/01.neu.0000168185.29659.c5

[10] Hellstrom P, Klinge P, Tans J, Wikkelso C. A new scale for assessment of severity and outcome in iNPH. Acta Neurol Scand. 2012;126(4)229–237. 10.1111/j.1600-0404.2012.01677.x 22587624

[11] Zetterberg M, Landgren S, Andersson ME, Palmér MS, Gustafson DR, Skoog I, et al Association of complement factor H Y402H gene polymorphism with Alzheimer's Disease. American Journal of Medical Genetics, Part B: Neuropsychiatric Genetics. 2008;147(6):720–726. 10.1002/ajmg.b.30668 18163432

[12] Blennow K, Ricksten A, Prince JA, Brookes AJ, Emahazion T, Wasslavik C, et al No association between the alpha2-macroglobulin (A2M) deletion and Alzheimer's disease, and no change in A2M mRNA, protein, or protein expression. Journal of neural transmission (Vienna, Austria: 1996). 2000;107(8–9):1065–1079. 10.1007/s007020070052 11041282

[13] Strandhagen E, Berg C, Lissner L, Nunez L, Rosengren A, Torén K, et al Selection bias in a population survey with registry linkage: potential effect on socioeconomic gradient in cardiovascular risk. European journal of epidemiology. 2010;25(3):163–172. 10.1007/s10654-010-9427-7 20127393

[14] Farrer LA, Cupples LA, Haines JL, Hyman B, Kukull WA, Mayeux R, et al Effects of age, sex, and ethnicity on the association between apolipoprotein E genotype and Alzheimer disease. A meta-analysis. APOE and Alzheimer Disease Meta Analysis Consortium. *JAMA*: *the journal of the* American Medical Association. 1997;278(16):1349–1356. 9343467

[15] Toma AK, Papadopoulos MC, Stapleton S, Kitchen ND, Watkins LD. Systematic review of the outcome of shunt surgery in idiopathic normal-pressure hydrocephalus. Acta Neurochir (Wien). 2013;155(10):1977–1980. 23975646 10.1007/s00701-013-1835-5

[16] Silverberg GD, Mayo M, Saul T, Fellmann J, Carvalho J, McGuire D. Continuous CSF drainage in AD: Results of a double-blind, randomized, placebo-controlled study. Neurology. 2008;71(3):202–209. 10.1212/01.wnl.0000316197.04157.6f 18525029

[17] Pyykko OT, Helisalmi S, Koivisto AM, Mölsä JA, Rummukainen J, Nerg O, et al APOE4 predicts amyloid-beta in cortical brain biopsy but not idiopathic normal pressure hydrocephalus. J Neurol Neurosurg Psychiatry. 2012;83(11):1119–1124. 10.1136/jnnp-2011-303849 22955176

[18] Jeppsson A, Zetterberg H, Blennow K, Wikkelso C. Idiopathic normal-pressure hydrocephalus: pathophysiology and diagnosis by CSF biomarkers. Neurology. 2013;80(15):1385–1392. 10.1212/WNL.0b013e31828c2fda 23486875

[19] Agren-Wilsson A, Lekman A, Sjoberg W, Rosengren L, Blennow K, Bergenheim AT, et al CSF biomarkers in the evaluation of idiopathic normal pressure hydrocephalus. Acta Neurol Scand. 2007;116(5):333–339. 17922727 10.1111/j.1600-0404.2007.00890.x

[20] Tarnaris A, Watkins LD, Kitchen ND. Biomarkers in chronic adult hydrocephalus. Cerebrospinal Fluid Research. 2006;3. 10.1186/1743-8454-3-11 PMC1617118 17020616

